# Random sampling of elementary flux modes in large-scale metabolic networks

**DOI:** 10.1093/bioinformatics/bts401

**Published:** 2012-09-03

**Authors:** Daniel Machado, Zita Soons, Kiran Raosaheb Patil, Eugénio C. Ferreira, Isabel Rocha

**Affiliations:** ^1^IBB-Institute for Biotechnology and Bioengineering/Centre of Biological Engineering, University of Minho, Campus de Gualtar, 4710-057 Braga, Portugal; ^2^Department of Bioinformatics and Functional Genomics, Institute of Pharmacy and Molecular Biotechnology, and Bioquant, University of Heidelberg, Im Neuenheimer Feld 267, 69120; ^3^Department of Theoretical Bioinformatics, German Cancer Research Center (DKFZ), Im Neuenheimer Feld 280, 69120; ^4^Structural and Computational Biology Unit, EMBL-Heidelberg, Meyerhofstrasse 1, 69117 Heidelberg, Germany

## Abstract

**Motivation:** The description of a metabolic network in terms of elementary (flux) modes (EMs) provides an important framework for metabolic pathway analysis. However, their application to large networks has been hampered by the combinatorial explosion in the number of modes. In this work, we develop a method for generating random samples of EMs without computing the whole set.

**Results:** Our algorithm is an adaptation of the *canonical basis approach*, where we add an additional filtering step which, at each iteration, selects a random subset of the new combinations of modes. In order to obtain an unbiased sample, all candidates are assigned the same probability of getting selected. This approach avoids the exponential growth of the number of modes during computation, thus generating a random sample of the complete set of EMs within reasonable time. We generated samples of different sizes for a metabolic network of *Escherichia coli*, and observed that they preserve several properties of the full EM set. It is also shown that EM sampling can be used for rational strain design. A well distributed sample, that is representative of the complete set of EMs, should be suitable to most EM-based methods for analysis and optimization of metabolic networks.

**Availability:** Source code for a cross-platform implementation in Python is freely available at http://code.google.com/p/emsampler.

**Contact:** dmachado@deb.uminho.pt

**Supplementary information:** Supplementary data are available at *Bioinformatics* online.

## 1 INTRODUCTION

The description of a metabolic network in terms of elementary (flux) modes (EMs) provides an important framework for metabolic pathway analysis (Schuster *et al.*, 1999). Elementary mode analysis identifies all minimal functional pathways connecting substrates with biomass and products inherent to a metabolic network. EMs have been used to understand the cellular metabolism through analysis of the network structure, regulations and characterization of all possible phenotypes (Çakir *et al.*, 2007; Schuster *et al.*, 1999, 2000, 2002; Stelling *et al.*, 2002). Examples of other recent applications of pathway analysis are in the determination of minimum medium requirements (Schilling and Palsson, 2000) and in the development of reduced kinetic models (Provost and Bastin, 2004). They also play an essential role in the development of model-based metabolic engineering strategies for strain optimization by identification of suitable intervention targets (Hädicke and Klamt, 2010, 2011; Trinh *et al.*, 2008). A comprehensive review on elementary mode analysis and other applications of EMs can be found in (Trinh *et al.*, 2009).

EMs are also closely related to the problem of identifying all transition invariants (t-invariants) in Petri net theory (Chaouiya, 2007). In fact, if all reactions are irreversible, the set of EMs is equivalent to the minimal t-invariants of a Petri net. Thus, it is not surprising that the algorithms for computation of EMs and t-invariants have evolved closely [see Schuster *et al.* (2002) for a comparison of both concepts].

Despite recent improvements in the algorithms for computation of EMs (Klamt *et al.*, 2005; Terzer and Stelling, 2008), their application to real world metabolic networks has been hampered by the combinatorial explosion in the number of modes as the size of the networks increase. The enumeration of the complete set of EMs for genome-scale networks has been infeasible so far, and perhaps even undesirable due to the hardly manageable number of modes that would be generated.

An attractive approach is the enumeration of a subset of pathways representing the complete system. Several approaches have been proposed hereto, though none of them provides a purely random sample of EMs. Current state of the art approaches typically enumerate EMs with a certain objective or constraint; like the enumeration of the shortest pathways (De Figueiredo *et al.*, 2009; Rezola *et al.*, 2011), the enumeration of pathways including a specific target reaction (Kaleta *et al.*, 2009b), enumeration based on available measurements (Jungers *et al.*, 2011; Soons *et al.*, 2011), enumeration of all possible pathways through selected reactions that satisfy the steady-state flux of the entire network (elementary flux patterns) (Kaleta *et al.*, 2009a), or decomposition of the network in modules (Schuster *et al.*, 2002; Schwartz *et al.*, 2007). These approaches do not represent the full solution space and hence a number of potentially interesting solutions may be missed.

One of the key requirements for successful understanding of the cellular metabolism based on EMs is the ability to enumerate a representative subset of modes. In this work, we develop a method for generating random samples of EMs without computing their whole set. The goal is to obtain a well-distributed sample, which is representative of the complete set of EMs, and suitable to most EM-based methods for analysis and optimization of metabolic networks.

## 2 METHODS

### 2.1 Algorithm

The EM sampler was implemented as an adaptation to the *canonical basis approach* by Schuster and Hilgetag (Schuster and Hilgetag, 1994). The algorithm begins with a matrix containing the transposed stoichiometric matrix augmented with the identity matrix. Then, for each metabolite, all the non-zero entries in the corresponding column are detected and replaced with all possible combinations that annul the respective entries. The combinatorial nature of the pairwise input/output reaction combination for each metabolite is the key to the exponential growth of the number of modes during the execution of the algorithm. Therefore, we add an additional filtering step which, at each iteration, selects only a subset of the possible combinations. This selection is based on a given probability function that randomly selects among the candidates. The algorithm is described as follows:

- initialize the matrix *T* = [*S^T^* |*I_n_*]
- for *i* ∈ {1,..., *n*} :
  **–** let *R* = {*j* | *T_j,i_* ≠= 0}
  **–** delete all rows *R* from *T*
  **–** let *T*_new_ = []
  **–** for (*j, k*) ∈ combinations(*R*):
    * *t* = add(*T_j,_*_•_, *T_k,_*_•_)
    * rev(*t*)= rev(*T_j,_*_•_) ∧ rev(*T_k,_*_•_)
    * if minimal(*t_m_*_+1:_*_m_*_+_*_n_*) : append *t* to *T*_new_
  **–** *T*_new_ = filter(*T*_new_)
  **–** append *T*_new_ to *T*
- get *E* from *T* = [0|*E^T^* ]

where *S* ∈ ℝ*^m^*^×^*^n^* is the stoichiometric matrix, *I_n_* is the identity matrix of size *n* and *E* is the elementary mode matrix. In order to find all possible row combinations, the following function is defined:

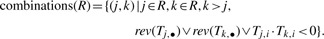


Function *rev* keeps track of the reversibility of the candidate modes so that reversible modes can be freely combined, and irreversible modes can only be combined in the appropriate direction. A candidate mode is reversible if it only contains reversible reactions. In order to combine candidate modes such that column *i* is annulled (i.e. metabolite *i* is balanced), it is important to make sure that they are combined in the proper direction. This is implemented by the following function:

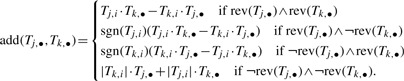


For a mode to be elementary, it must have minimal support (Schuster *et al.*, 1999). There are two methods to check if a mode is elementary, the *combinatorial test* and the *rank test* (Terzer and Stelling, 2008). The combinatorial test compares the support vector of the new mode with the support of all modes computed at that point. However, this method is not appropriate in our case because we do not have the full set of elementary modes required for the test. On the other hand, the rank test is based only on the support vector of the candidate mode. Therefore, we opted to use this test in our implementation:

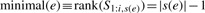

where *s*(*e*)= {*i* | *e_i_* ≠ 0} is the support of *t*, and *S*_1:_*_i,s_*_(_*_e_*_)_ is a submatrix of the stoichiometric matrix composed by the metabolites that have been processed so far, and the reactions that belong to the support of *e*. We also verify if the new candidate mode contains any reversible reaction occurring in both directions simultaneously. In that case, the candidate can be disregarded without performing the test.

**Fig. 1.**
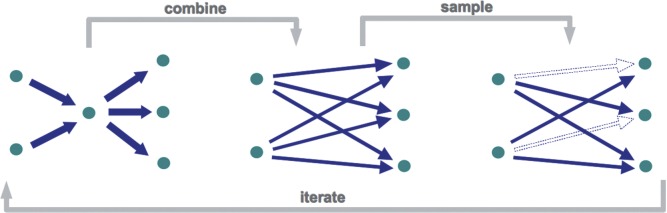
The computation of elementary modes consists on iteratively removing all internal metabolites, and combining every pair of input/output reactions. For highly connected nodes, this results in a combinatorial explosion of new connections (expansion phase). To avoid the exponential growth along the iterations, caused by the accumulation of this effect, we add an additional filtering step that randomly samples the new combinations (contraction phase)

The filtering step is the novelty of the method proposed in this work (Fig. 1). In order to prevent the exponential growth in the number of candidate modes, we randomly select a sample of the new candidate modes at each step. This is implemented by the following function:

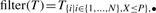

where *N* is the number of new candidate modes, 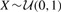 and *P* is a given selection probability.

The selection probability is a critical aspect of the algorithm. A low probability may cause the elimination of vital connections in the network, whereas a high probability may not prevent the combinatorial explosion. Ideally, one would want a high selection probability for low connectivity nodes, and a low selection probability at high connectivity nodes. Therefore, we opted to define the selection probability as a function of the number of candidate modes. Furthermore, we can observe that the selection of modes follows a binomial distribution with an average selection size equal to *N* ·*P*. Hence, at each step we define 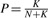 such that:

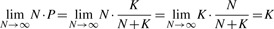

where *K* is a given constant that determines a maximum upper bound in the number of new candidate modes at each step.

### 2.2 Implementation

The algorithm was implemented in Python and uses the open libraries NumPy/SciPy (Jones *et al.*, 2012) for numerical computations, and libSBML (Bornstein *et al.*, 2008) for reading SBML (Systems Biology Markup Language) model files. All tests were performed on an Intel Core 2 Duo 2.13 GHz processor with 3 GB RAM, running Linux Kernel 3.0 and Python 2.7.

### 2.3 Model

The algorithm was tested using a condensed genome-scale metabolic reconstruction of *Escherichia coli* (Orth *et al.*, 2009). The model contains 72 metabolites (52 internal, 20 external) and 95 reactions (75 internal, 20 drains). Glucose was set as the external carbon source and flux variability analysis (FVA) (Mahadevan and Schilling, 2003) was performed in order to detect blocked reactions, which were then removed from the model. The simplified model contains 68 metabolites and 87 reactions. The EMs for this model were calculated with *efmtool* (Terzer and Stelling, 2008), resulting in a total of 100.274 EMs.

## 3 RESULTS

### 3.1 Sampling

The selection probability is controlled by the constant *K*, the only adjustable parameter in the algorithm (see Section 2). Therefore, we performed several tests using different values for this parameter. For each value of *K*, a total of 10 trials were run, and the individual samples were merged into a larger set. Additionally, we verified that all modes obtained are truly elementary modes contained within the full set of EMs for the tested model.

**Fig. 2.**
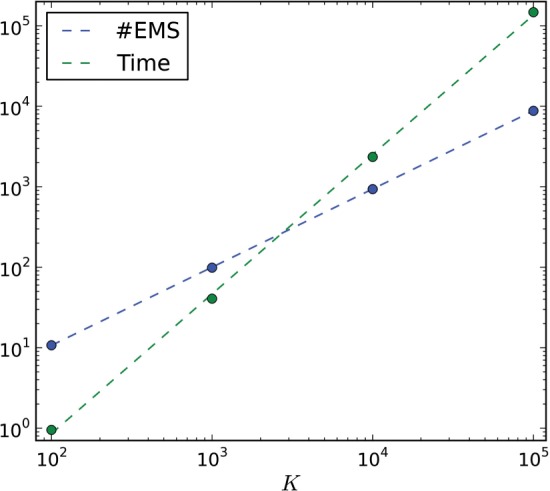
Sample size (No EMs) and computation time (s) as a function of *K* (log–log scale). The number of EMs grows linearly with *K* (slope ≃ 1.0), whereas the computation time grows nearly quadratically (slope ≃ 1.7)

The first test is the sample size and computation time as a function of *K*. By controlling the selection probability, it is expected that the resulting sample size will be affected and, consequently, the computation time as well. Results for these experiments are shown in Table 1. By performing linear regression of these values on a log–log scale, it is possible to observe that the number of modes obtained grows linearly with *K*, whereas the computation time grows nearly quadratically (Fig. 2).

**Table 1.** Size of the EM samples obtained and respective computation times for different values of K

K	No EMs	Time (s)
10^2^	(1.1±0.6)×10^1^	(9.5±4.9)×10^−1^
10^3^	(9.9±3.3)×10^1^	(4.1±1.7)×10^1^
10^4^	(9.3±0.6)×10^2^	(2.4±0.8)×10^3^
10^5^	(8.8±0.2)×10^3^	(1.5±0.2)×10^5^

The data represent the mean values and standard deviation for 10 trials per experiment.

**Fig. 3.**
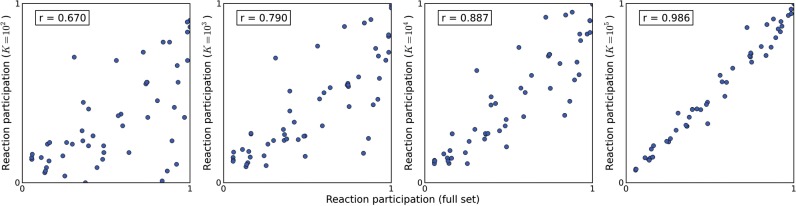
Reaction participation in the full EM set versus the participation in samples of different sizes, and the respective Pearson correlation coefficients (*r*)

In order to obtain a well-distributed sample it is important to guarantee that the reaction participation (i.e. the fraction of EMs in which a reaction participates) is preserved by the sampling procedure. Otherwise we would obtain a biased sample of the full EM set. We compared the reaction participation of the generated samples against the respective values in the full set of EMs (Fig. 3). It is possible to observe that for lower values of *K*, there is a weaker correlation between the reaction participation of the samples and the reaction participation of the full EM set. This is likely due to the fact that the sample size is too small to obtain a good coverage of the solution space. However, it is possible to observe that, as *K* increases, the Pearson correlation coefficient (*r*) also increases. For *K* = 10^5^ we observe a high correlation (*r* = 0.986) between the reaction participation that is estimated by the sampling approach and the true values. In all cases, the dispersion seems to be homogeneous, showing no observable bias, hence the degree of correlation is only affected by the sample size.

**Fig. 4.**
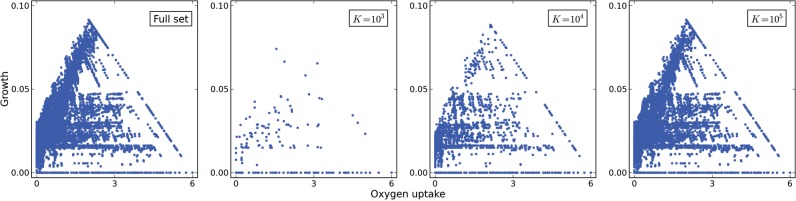
Comparison of the phenotypic phase planes for oxygen uptake and cellular growth normalized by glucose uptake (full set and different samples)

Furthermore, we analyzed the EM samples regarding their distribution within the flux solution space. For that matter, we plotted the EMs distribution within the phenotypic phase plane for oxygen uptake and cellular growth normalized by glucose uptake (Edwards *et al.*, 2002). Figure 4 shows the distribution for the full set compared with the samples for different values of *K*. It can be observed that the samples are unbiased relatively to the full set, and that the coverage of the solution space improves with the size of the samples.

**Fig. 5.**
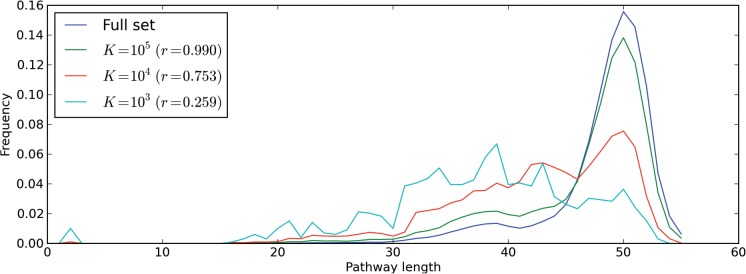
Comparison of the pathway length distribution of the full set of EMs against the distribution for samples at different values of *K* and the correlation coefficient (*r*) between the original frequency distribution and the latter

We also evaluated how the sampling procedure affects the pathway length distribution (Fig. 5). It is possible to observe that the full EM set has a skewed Gaussian-like distribution with a maximum frequency of pathways with 50 reactions. However, we observe that as *K* decreases, the distribution shifts towards smaller pathway lengths. This is not surprising since the EMs with larger support vectors will undergo more sampling steps. Nonetheless, it is observed that for *K* = 10^5^, the distribution of the sample is considerably close to the distribution of the full set.

### 3.2 Case study: rational strain design

One of the applications of elementary mode analysis is the rational design of mutant strains for industrial production of chemical compounds (Hädicke and Klamt, 2010, 2011; Trinh *et al.*, 2008). The enumeration of the EMs of a metabolic network, allows the determination of the most efficient pathways for production of the selected compound. Then, one can find the best knockout candidates that eliminate the maximum number of competing pathways, channeling the metabolic fluxes to the desired pathways (Trinh *et al.*, 2008).

In order to understand if the utilization of EM samples is appropriate for rational strain design, we compared the knockout strategy obtained with the full EM set, using the method of (Trinh *et al.*, 2008), against the strategies obtained with different samples (for *K* = 10^5^). Succinate production was used as the case study, all experiments were constrained to a maximum of 8 knockouts, and only strategies with viable biomass production were allowed. In order to have an estimate of the production rate for each case, we used the method of minimization of metabolic adjustment (MOMA) to predict the flux distribution of the mutants (Segrè *et al.*, 2002).

The results are presented in Table 2. It is possible to observe that the knockout strategies found for the samples differ from that of the full EM set (see Supplementary Fig. S1 for a clustering analysis). Nonetheless, all the knockouts predicted with the full set appear frequently in the other knockout strategies. Also, the succinate production rates estimated with MOMA are high in most cases. Only in one case (Sample 8) there was no production, although it predicted 4 knockouts common to the full EM set.

**Table 2.** Comparison of the optimal knockout strategies for succinate production for the full EM set and different EM samples

Test	No Total EMs (suc)	Reaction knockouts	No EMs (suc)	Est. rate
Full EM set	100 273 (48 602)	**ACALD**, **G6PDH2r**, **GLUDy**, **LDH_D**, **NADTRHD**, **PFL**, **PYK**, **SUCDi**	406 (320)	6.897
Sample 1	8745 (3962)	**ACALD**, GLUSy, ICL, **LDH_D**, **PFL**, PGI, **SUCDi**, THD2	59 (34)	3.397
Sample 2	9001 (3979)	**ACALD**, GLUSy, ICL, **LDH_D**, ME2, **PFL**, PGI, **SUCDi**	69 (30)	3.397
Sample 3	8607 (4011)	**ACALD**, ATPS4r, **GLUDy**, ME1, **PFL**, **PYK**, **SUCDi**, TKT2	50 (37)	4.435
Sample 4	8682 (3838)	**ACALD**, ACKr, **GLUDy**, ICL, **LDH_D**, **PFL**, RPE, **SUCDi**	48 (42)	6.647
Sample 5	8489 (3553)	**ACALD**, ACKr, GLUSy, **LDH_D**, **PFL**, PGI, **SUCDi**, THD2	76 (52)	3.473
Sample 6	8453 (3574)	ATPS4r, GLUSy, ME1, **NADTRHD**, **PFL**, **PYK**, **SUCDi**, TALA	44 (36)	2.004
Sample 7	9056 (4080)	**ACALD**, GLUSy, ME1, ME2, **PFL**, PGI, **SUCDi**, THD2	81 (55)	3.473
Sample 8	8877 (4228)	**ACALD**, ATPS4r, FBP, **G6PDH2r**, GLUSy, ME1, **NADTRHD**, **PFL**	38 (10)	0.000
Sample 9	8647 (4007)	**ACALD**, **G6PDH2r**, GLUSy, ME2, **NADTRHD**, **PFL**, **PYK**, **SUCDi**	41 (30)	6.899
Sample 10	9097 (4129)	**ACALD**, ATPS4r, GLUSy, **LDH_D**, **NADTRHD**, **PFL**, **SUCDi**, TALA	41 (31)	0.207

Total number of EMs (succinate producing); Optimal reaction knockouts; Number of remaining EMs (succinate producing); Estimated production rate (mmol/gDW/h) computed from MOMA.

## 4 DISCUSSION

### 4.1 Sampling quality

The main goal of this work is the creation of a sampling approach for computing EMs in large-scale networks without computing their whole set. For that matter, it is important to guarantee that the sampling approach provides a uniform coverage of the complete solution space. Our method is controlled by a single parameter that influences the number of computed EMs, by adjusting the selection probability during the execution of the algorithm. Our results show that the sample size obtained is directly proportional to *K*.

For most EM-based applications, it is important to obtain a sample that preserves the reaction participation (the fraction of EMs in which a reaction participates). The results show that the sampling is unbiased in that aspect. However, the correlation of the estimated values with the true values is affected by the size of the sample. The larger the sample size, the better will be the correlation obtained. This is also reflected in the analysis of the phenotypic phase plane for oxygen uptake and cellular growth. The samples present an unbiased representation of the solution space, although the coverage obtained will depend on the sample size.

Regarding the pathway length distribution of the EMs, the results show that there can be a bias towards smaller pathway lengths for low values of *K*. The importance of this bias depends on the application for which the sample will be used. One may argue that shorter pathways are more efficient, hence more likely to carry higher fluxes. For a large value of *K*, we can observe that the bias is not significant. However, for larger networks, the demands in terms of computational time and memory may not allow for arbitrarily, large values of *K* and the effect may become more significant. One way to compensate for such effect would be to give larger selection probabilities to modes with larger support vectors. In that case, additional testing is required in order to check if that artificial selection would cause any bias in other properties of the samples.

We tested our approach with a case study of rational strain design for succinate production in *E.coli*. The results have shown that, using an EM sample, it is possible to predict most of the best potential reaction knockouts, and to obtain close to optimal solutions. The utilization of heuristic methods to search for satisfactory solutions, is a common approach in metabolic engineering for large metabolic networks, when an exhaustive search becomes prohibitive (Patil *et al.*, 2005).

### 4.2 Performance

We implemented our sampling method as an adaptation to the *canonical basis approach* (Schuster and Hilgetag, 1994). This approach has a very simple and intuitive topological interpretation in terms of the graph of the metabolic network (Fig. 1). However, it is very inefficient compared to the more recent *nullspace approach* (Klamt *et al.*, 2005). There are very efficient implementations of this approach (e.g. using *bit pattern trees* (Terzer and Stelling, 2008)). The *efmtool* software, which implements these state-of-the-art methods (Terzer and Stelling, 2008), is able to compute the full set of approximately a hundred thousand EMs in the order of seconds to minutes. Our implementation of the *canonical basis approach*, on the other hand, takes within minutes to hours to compute a few thousand EMs. In our tests, we used a condensed genome-scale reconstruction of *E. coli* (Orth *et al.*, 2009), which is a simplified version of the full genome-scale model (Feist *et al.*, 2007). In order to apply our method to the full model, it will be necessary to analyze how this approach can be reformulated as a modification to the *nullspace approach* and integrated into the most recent implementations (Terzer and Stelling, 2008).

The most significant bottleneck in our algorithm is the computation of a matrix rank for every candidate mode. As explained earlier (see Section 2), the *combinatorial test* is not appropriate for EM sampling because we do not have the full set of EMs to compare with. Using this method in our approach would result in the computation of a sample of modes that are elementary among themselves but not truly elementary modes of the full set. Therefore, the *rank test* must be used. However, computing the rank of a large matrix is very expensive and hampers EM computation at the genome-scale. This limitation may be overcome by improving the efficiency of rank calculation. Note that we are constantly computing the rank of matrices which are very similar (submatrices of the stoichiometric matrix). Therefore, one may take advantage of methods with pre-computation such as the *lazy rank updating* method proposed by (Terzer and Stelling, 2008).

Our results show that the computational time grows nearly quadratically with the size of the sample. Therefore, it would seem advantageous to use a divide-and-conquer strategy to compute a sample of size *N* by appending together *P* independent samples of size *N/P*. However, in order to obtain smaller sample sizes, one has to decrease the selection probability (by adjusting *K*), which affects the quality of the samples regarding the pathway length distribution. Note that it is possible to take advantage of multiple CPUs to run several samplers in parallel and combine the samples into one larger set. However, it must be kept in mind that this does not provide the same sampling quality as a sample of the same size obtained with a higher selection probability.

One of the advantages of elementary mode analysis, when compared with methods based on flux balance analysis, is the fact that the EM set needs to be computed only once. Once the EM set is computed, the analysis and optimization of the metabolic network is quite straightforward. On the other hand, bi-level optimization frameworks require expensive computational time at every utilization (Burgard *et al.*, 2003; Patil *et al.*, 2005). Therefore, even if the computation of an EM sample, large enough to obtain an unbiased coverage of the solution space, is highly time consuming at the genome-scale, this effort is compensated on the long term.

## 5 CONCLUSION

As more data are collected, metabolic models keep constantly growing in size. This increases the challenge for EM-based analysis of metabolic networks, as the number of EMs grows exponentially with the network size. For that matter, the development of EM sampling approaches will become increasingly important. This work is a contribution in that direction. We developed a method that prevents the combinatorial explosion of the number of EMs during computation, by adding a filtering step that randomly samples among the candidate modes at each iteration. Unlike other methods for obtaining reduced sets of EMs (De Figueiredo *et al.*, 2009; Jungers *et al.*, 2011; Kaleta *et al.*, 2009b; Rezola *et al.*, 2011), our approach does not use any objective functions or experimental flux constraints.

EFMEvolver (Kaleta *et al.*, 2009b) is the approach most similar to ours. It samples the EMs that contain a target reaction, rather than the whole solution space. It uses linear programming (LP) to find a single EM, and a genetic algorithm (GA) to search different solutions. It has the advantage that the procedure can be stopped after a desired number of modes have been collected, whereas our approach only yields valid EMs after completion. On the downside, it requires tuning the parameters for the GA and selection of a proper fitness function, whereas or method is tunable by a single parameter. Our method can show a bias towards smaller EM pathway lengths if the selection probability is too low. Given its formulation, it is likely that EFMEvolver exhibits the same bias, although it is not evaluated how strong that bias can be.

Despite the current shortcomings, EM sampling is a promising approach for computation of EMs at the genome-scale, and opens the possibility for application of EM-based metabolic engineering methods for optimizing metabolic networks at this scale.

*Funding:* Research supported by the Portuguese Foundation for Science and Technology (FCT), through the projects “Bridging Systems and Synthetic Biology for the development of improved microbial cell factories” (MIT-Pt/BS-BB/0082/2008) and “SYNBIOBACTHER - Synthetic biology approaches to engineer therapeutic bacteria” (PTDC/EBB-BIO/102863/2008).

*Conflict of Interest:* none declared.
